# Candidate Screening for Heart Failure With Preserved Ejection Fraction Clinic by Fib-4 Index From Subclinical Subjects

**DOI:** 10.1016/j.gastha.2022.09.005

**Published:** 2022-09-21

**Authors:** Chisato Okamoto, Osamu Tsukamoto, Takuya Hasegawa, Tatsuro Hitsumoto, Ken Matsuoka, Makoto Amaki, Hideaki Kanzaki, Chisato Izumi, Seiji Takashima, Shin Ito, Masafumi Kitakaze

**Affiliations:** 1 Department of Medical Biochemistry, Osaka University Graduate School of Medicine/Frontier Biosciences, Suita, Osaka, Japan; 2 Department of Clinical Medicine and Development, National Cerebral and Cardiovascular Center, Suita, Osaka, Japan; 3 Department of Cardiovascular Medicine, Garatia Hospital, Mino, Osaka, Japan; 4 Department of Cardiovascular Medicine, National Cerebral and Cardiovascular Center, Suita, Osaka, Japan; 5 Department of Cardiovascular Medicine, Hanwa Memorial Hospital, Osaka, Osaka, Japan

**Keywords:** Heart-liver axis, Liver stiffness, HFpEF, Mass screening

## Abstract

**Background and Aims:**

Recognition of heart failure with preserved ejection fraction (HFpEF) at an early stage in mass screening is desirable, but difficult to achieve. We examined whether the fibrosis (Fib)-4 index, a simple index of liver stiffness/fibrosis, could be used as a screening tool to select candidates requiring expert diagnostics.

**Methods:**

Individuals who participated in annual health checks between 2006 and 2007 in Arita-cho, Saga, Japan, with no history of cardiovascular disease and EF ≥ 50% were enrolled (total 710; 258 men; median age, 59 years).

**Results:**

Participants were divided into 5 groups according to HFpEF risk: 215 (30%), 100 (14%), 171 (24%), 163 (23%), and 61 (9%) with Heart Failure Association (HFA)-PEFF scores of 0, 1, 2, 3, and 4–6 points, respectively. The highest HFpEF risk group (HFA-PEFF score, 4–6 points) showed poor prognosis for the clinical events of all-cause mortality and hospitalization for HF (log-rank test, *P* = .002). The Fib-4 index was correlated with HFpEF risk stratification (r_s_ = 0.526), and increment in the Fib-4 index was independently linked to high HFpEF risk by multiple logistic regression analysis (adjusted odds ratio, 1.311; 95% confidence interval, 1.078–1.595; *P* = .007). The Fib-4 index stratified clinical prognosis (log-rank test, *P* < .001) was an independent predictor of all-cause mortality and hospitalization for HF (hazard ratio, 1.305; 95% confidence interval, 1.139–1.495; *P* < .001).

**Conclusion:**

The Fib-4 index can be used to select appropriate candidates for a detailed examination of HFpEF in a subclinical population.

## Introduction

Heart failure with preserved ejection fraction (HFpEF), an emerging disease in recent years,^1^ has a poor prognosis similar to HF with reduced EF (HFrEF)^2^ and a higher rate of noncardiovascular events than HFrEF.^3^ Recognition of HFpEF at an early stage in a subclinical population during mass screenings, such as health check-up programs, for therapeutic intervention seems to be desirable because even a single HF episode that requires hospitalization results in poor prognosis.^4^ Additionally, favorable medication therapies have been reported in recent years.^5^, ^6^, ^7^ However, diagnosis of HFpEF at an early stage is challenging; although the current standard definition of HFpEF is based on the clinical symptoms of HF and objective evidence of spontaneous or provokable elevated left ventricular (LV) filling pressures without LV systolic dysfunction,^8^, ^9^, ^10^ HFpEF patients are often asymptomatic until late in the disease process^11^ and have normal LV filling pressures at rest.^12^^,^^13^ If we have a simple and objective tool to select probable cases with HFpEF from subclinical participants in a health check-up program, we can refer them to a specialized hospital to obtain a diastolic stress test, the gold standard for the diagnosis of HFpEF,^14^^,^^15^ which may help in the early diagnosis of HFpEF. The Heart Failure Association (HFA)–PEFF diagnostic algorithm was developed to optimize the diagnosis of HFpEF in patients with breathlessness; the HFA-PEFF score is used to assess HFpEF risk and determine whether to perform a stress test.^16^ However, it requires echocardiographic measurements,^16^ which require special equipment and expertise.

HF and liver stiffness/fibrosis often coexist due to cardio–hepatic interactions. Increased central venous pressure (CVP) due to HF is related to liver stiffness, resulting in fibrosis and adverse prognosis.^17^^,^^18^ Indeed, the fibrosis (Fib)-4 index, a simple and validated noninvasive screening tool for liver stiffness/fibrosis, is associated with a higher risk of major adverse cardiovascular events (MACE) in patients with HFpEF.^19^^,^^20^ However, the liver can also cause cardiac dysfunction. Nonalcoholic fatty liver disease (NAFLD) is the most common chronic liver condition affecting a quarter of adults worldwide,^21^ in which hepatic steatosis and inflammation accompanied with liver stiffness/fibrosis are commonly observed. Patients with NAFLD have a two-fold higher prevalence of HFpEF than the general population.^22^ In addition, excessive salt in the diet appears to induce hepatic steatosis and inflammation, similar to NAFLD, which contributes to cardiovascular damage.^23^ These studies suggest that diet-induced hepatic inflammatory memory with liver stiffness/fibrosis, as reflected by the Fib-4 index, contributes to the development of HFpEF in the general population.

Therefore, we hypothesized that the Fib-4 index could be an objective and useful screening tool for selecting candidates from the subclinical population who require specialized diagnostic studies for the early diagnosis of HFpEF. To test this hypothesis, we examined the association between the Fib-4 index and HFpEF risk assessed by the HFA-PEFF score and investigated the prognostic impact of the Fib-4 index in a large subclinical population attending health check-up programs.

## Materials and Methods

### Study Population

A total of 822 individuals who participated in the health check-up program of Arita-cho, Saga, Japan, between 2006 and 2007 (the Arita-cho cohort study) were enrolled in this study.^24^^,^^25^ Participants with a history of cardiovascular diseases such as HF, atrial fibrillation, angina, myocardial infarction, cardiovascular surgery, pacemaker implantation, and/or valvular disease at baseline were excluded from the follow-up. We included patients with no deficient parameters for the LV mass index (LVMI), relative wall thickness (RWT), and septal E/e’ calculated from transthoracic echocardiography, with measures for B-type natriuretic peptide (BNP), aspartate aminotransferase (AST), alanine aminotransferase (ALT), and platelet counts. We excluded patients with an LV ejection fraction (LVEF) < 50%. Ultimately, 710 individuals were included in the analysis. This study was conducted in accordance with the Declaration of Helsinki and approved by the ethics committees of the National Cerebral and Cardiovascular Center, Arita-cho (M28-077-3). Written informed consent was obtained from all participants before their participation in the study.

### Measures

Blood samples were collected at least 10 hours after the last food intake, and the Fib-4 index was calculated using the following formula^26^:

*age (years) × AST [U/L]/(platelet [10*^*9*^*/L]) × (ALT [U/L]*^*1/2*^*)*.

Echocardiographic assessments were performed in accordance with the American Society of Echocardiography guidelines.^27^^,^^28^ LV end-diastolic and end-systolic diameters (LVDd and LVDs, respectively), interventricular septal thickness (IVST), LV posterior wall thickness (PWT), peak E wave, and septal e’ velocities were measured. Both RWT and LV mass were calculated according to the American Society of Echocardiography guidelines; LVMI was calculated by correcting for the body surface area:

*LVMI = RWT [mm] = (IVST + PWT)/LVDd, LVM [g] = 0.8 (1.04[{LVDd + IVST + PWT}3 – {LVDs}3]) + 0.6*.

We calculated the HFA-PEFF score using the algorithm proposed by Pieske et al., which assigns 2 points for a major criterion and one point for a minor criterion within each functional, morphological, and biomarker domain as follows: 2 points for either septal e' < 7 cm/s or septal E/e' ≥ 15, 1 point for septal E/e' 9–14 for the functional domain, 2 points for both LVMI ≥ 149/122 g/m^2^ (m/w) and RWT > 0.42, 1 point for either LVMI > 115/95 g/m^2^ or RWT > 0.42, or LV wall thickness ≥12 mm for the morphological domain, and 2 points for BNP > 80 pg/ml, and 1 point for BNP 35–80 pg/ml for the biomarker domain.^16^

Information on disease history was obtained using a standardized questionnaire at baseline and during the annual health check-ups. The composite endpoints of all-cause mortality and hospitalization for HF were evaluated as clinical events, whereas the composite endpoints of cardiovascular mortality, occurrence of acute myocardial infarction and stroke, hospitalization for HF, and ischemic cardiovascular events were evaluated as MACE. The development of liver cancer was evaluated as a liver-related outcome. For participants without clinical events, the final follow-up date was the date of the last contact.

### Statistical Analysis

Values are expressed as mean ± standard deviation if the variable is normally distributed, or as median (interquartile range) if not. Groups were compared using Student’s t-test, Wilcoxon test, or Kruskal–Wallis test for continuous variables and chi-squared test for categorical variables, as appropriate. The Shapiro–Wilk test was used to assess whether the data were normally distributed. All tests were two-sided, and statistical significance was set at *P* < .05. The correlation between the Fib-4 index and various parameters was evaluated using Pearson’s correlation coefficient or Spearman’s rank correlation coefficient. Multiple logistic regression analysis was used to investigate the factors associated with the Fib-4 index and diastolic dysfunction, and adjusted odds ratios (aORs) and 95% confidence intervals (CIs) were calculated. Factors that were biologically essential and considered to be associated with the outcomes were included in the multivariate analyses as potential confounders, and there were no missing data. We utilized 3 multivariate models to confirm the robustness of the results. Receiver operating characteristic curve analysis was used to verify the validity of the cutoff value of the Fib-4 index for predicting high HFpEF risk. Kaplan–Meier analysis was used to evaluate clinical events during follow-up, and differences in survival curves were tested using a log-rank test (Mantel–Cox test). Cox proportional hazards analysis was performed to evaluate the influence of the Fib-4 index on events. Variables with statistical significance in univariate analysis were included in the multivariate models. Multicollinearity among the variables in the model was assessed by calculating the variance inflation factor and correlation coefficient. All statistical analyses were performed using IBM SPSS Statistics, Version 26.0. (IBM Corp: Armonk, NY, USA).

## Results

### HFpEF Risk Assessed by the HFA-PEFF Score in Subclinical Population for Clinical Prognosis

Data from 710 participants, without heart disease or reduced LV systolic function (LVEF ≥ 50%), from the Arita-cho health check-up program, were analyzed. First, we calculated the HFA-PEFF score to confirm whether HFpEF risk assessed using the HFA-PEFF score is associated with clinical prognosis in this subclinical cohort. Table 1 lists the results of 215 (30%), 100 (14%), 171 (24%), 163 (23%), 43 (6%), 14 (2%), and 4 (1%) patients with HFA-PEFF scores of 0, 1, 2, 3, 4, 5, and 6 points, respectively.

**Table 1:** Calculation of the HFA-PEFF Score

HFA-PEFF score	All cohort N = 710	Functional domain			Morphological domain			Biomarker domain		
		0 points	1 point	2 points	0 points	1 point	2 points	0 points	1 point	2 points
0 point (%)	215 (30)	215 (100)	0 (0)	0 (0)	215 (100)	0 (0)	0 (0)	215 (100)	0 (0)	0 (0)
1 point (%)	100 (14)	58 (58)	42 (42)	0 (0)	55 (55)	45 (45)	0 (0)	87 (87)	13 (13)	0 (0)
2 points (%)	171 (24)	9 (5)	18 (11)	144 (84)	152 (89)	18 (11)	1 (1)	156 (91)	14 (8)	1 (1)
3 points (%)	163 (23)	1 (1)	4 (2)	158 (97)	42 (26)	120 (74)	1 (1)	119 (73)	41 (25)	3 (2)
4 points (%)	43 (6)	0 (0)	1 (2)	42 (98)	7 (16)	32 (74)	4 (9)	4 (9)	31 (72)	8 (19)
5 points (%)	14 (2)	0 (0)	0 (0)	14 (100)	0 (0)	11 (79)	3 (21)	0 (0)	3 (21)	11 (79)
6 points (%)	4 (1)	0 (0)	0 (0)	4 (100)	0 (0)	0 (0)	4 (100)	0 (0)	0 (0)	4 (100)

Next, participants with HFpEF risks were stratified into 3 groups according to the HFA-PEFF score: low-risk (0–1), intermediate-risk (2–3), and high-risk (4–6). The clinical characteristics of patients are summarized in Table 2. Although the HFA-PEFF score of 4 was originally classified as an intermediate score, we reclassified this as a high-risk score because it has been reported to carry a worse prognosis than scores of 0–3 in the subclinical population.^29^ Among those with intermediate and high HFpEF risk, over 90% had a functional domain of 2 points, indicating that participants with intermediate and high scores were mainly stratified by morphological and biomarker domains. Twenty-five (4%) patients had liver disease; 4 (0.6%) had hepatitis C; however, details of other liver diseases were not available. The prevalence of hepatitis C in this cohort was comparable to that estimated for hepatitis C virus at a carrier rate of 0.6% in Japan.^30^ Among the 3 groups, there were no significant differences in the prevalence of smoking, or diabetes mellitus, or pulse rate. Participants in the high HFpEF risk group were older; the majority were men; had higher blood pressure; and a greater prevalence of alcohol intake, hypertension, dyslipidemia, and liver diseases than those in the other groups. Echocardiographic LVEF and LV dimensions (end-diastolic and end-systolic) were not significantly different between the 3 groups. However, the high-risk group had a higher LVMI, larger left atrial diameter, thicker RWT, lower septal e’, and higher septal E/e’, all of which suggest elevated LV end-diastolic pressure due to LV diastolic dysfunction. Regarding laboratory examinations, there were differences in tests related to liver and renal functions (AST, ALT, estimated glomerular filtration rate, and platelet count), glucose intolerance (hemoglobin A1c), lipid profile (total/high-density lipoprotein/low-density lipoprotein cholesterol and triglyceride), and plasma BNP levels among the 3 groups. The Fib-4 index showed a stepwise increase across the 3 groups: 0.94 (0.74–1.34), 1.45 (1.16–1.88), and 1.99 (1.43–2.49) in the low-, intermediate-, and high-risk groups, respectively.

**Table 2:** Patient Characteristics Classified by HFpEF Risk Assessed Using the HFA-PEFF Score

Characteristics	All cohort N = 710	Low (0–1) N = 315	Intermediate (2–3) N = 334	High (4–6) N = 61	*P* Value
Age, years	59 (46–67)	46 (38–57)	65 (59–69)	69 (66–73)	<.001
Male, n (%)	258 (36)	95 (30)	140 (42)	23 (38)	.008
Body mass index, kg/m^2^	22.0 (20.1–24.1)	21.2 (19.5–23.5)	22.4 (20.6–24.3)	22.7 (20.4–25.3)	<.001
Smoke (%)					.087
Current	117 (16)	57 (18)	52 (16)	8 (13)	
Past	97 (14)	33 (10)	50 (15)	14 (23)	
None	496 (70)	225 (71)	232 (69)	39 (64)	
Alcohol intake (%)					<.001
Every day	153 (22)	48 (15)	87 (26)	18 (30)	
More than 60 g/d of alcohol	17 (2)	5 (2)	9 (3)	3 (5)	
Sometimes	213 (30)	119 (38)	85 (25)	9 (15)	
Never	344 (48)	148 (47)	162 (49)	34 (56)	
Systolic blood pressure, mmHg	128 (113–141)	116 (107–128)	134 (123–146)	147 (133–162)	<.001
Diastolic blood pressure, mmHg	79 (71–87)	74 (67-81-)	82 (76–91)	87 (79–94)	<.001
Pulse rate, beats/min	63 (58–70)	63 (58–70)	64 (58–71)	62 (55–68)	.195
Medical history					
Hypertension, n (%)	118 (17)	19 (6)	75 (22)	24 (39)	<.001
Dyslipidemia, n (%)	53 (8)	11 (3)	34 (10)	8 (13)	.001
Diabetes mellitus, n (%)	37 (5)	12 (4)	21 (6)	4 (7)	.323
Liver disease, n (%)	25 (4)	6 (2)	14 (4)	5 (8)	.034
Viral hepatitis (hepatitis C), n (%)	4 (1)	0 (0)	4 (12)	0 (0)	.104
Laboratory data					
AST, IU/L	21 (18–26)	20 (17–24)	22 (19–27)	24 (20–32)	<.001
ALT, IU/L	18 (14–25)	17 (12–23)	19 (15–25)	19 (15–27)	.004
Hemoglobin A1c, %	5.6 (5.4–5.8)	5.5 (5.2–5.7)	5.7 (5.5–6.0)	5.7 (5.6–6.1)	<.001
Total cholesterol, mg/dL	210 (186–232)	203 (181–224)	214 (190–239)	210 (186–230)	<.001
Triglyceride, mg/dL	89 (62–124)	77 (54–107)	97 (72–138)	98 (65–133)	< .001
HDL-cholesterol, mg/dL	63 (54–74)	65 (56–76)	61 (52–72)	63 (54–74)	.011
LDL-cholesterol, mg/dL	123 (102–145)	116 (97–139)	130 (107–152)	120 (103–139)	<.001
Estimated GFR, mL/min	90.2 (76.1–100.8)	92.7 (83.0–104.7)	88.5 (74.7–96.5)	84.7 (73.1–97.2)	<.001
Hemoglobin, g/dL	13.7 ± 1.5	13.6 ± 1.5	13.9 ± 1.5	13.6 ± 1.4	.005
Platelet count, 10^4^/μL	22.4 (19.3–25.8)	22.9 (19.8–26.2)	22.3 (19.1–25.6)	20.6 (17.1–23.5)	.001
Brain natriuretic peptide, pg/mL	16.5 (8.0–30.8)	10.1 (5.2–19.0)	19.3 (10.5–32.2)	57.0 (44.0–104.3)	<.001
Fib-4 index	1.29 (0.92–1.74)	0.94 (0.74–1.34)	1.45 (1.16–1.88)	1.99 (1.43–2.49)	<.001
Fib-4 index as categorical variables					<.001
Low (≤ 1.30)	366 (52)	228 (72)	129 (39)	9 (15)	
Intermediate (1.30–2.67)	297 (42)	80 (25)	178 (53)	39 (64)	
High (≥ 2.67)	47 (7)	7 (2)	27 (8)	13 (21)	
Echocardiographic parameters					
Interventricular septal thickness, mm	8.0 (7.0–9.4)	7.9 (7.0–9.0)	9.0 (8.0–10.0)	10.0 (9.0–11.2)	<.001
Posterior wall thickness, mm	9.0 (8.0–10.0)	8.0 (7.0–9.0)	9.0 (8.0–10.0)	10.0 (9.0–11.0)	<.001
LV end-diastolic dimension, mm	46.0 (43.0–49.0)	46.0 (43.0–49.0)	46.0 (43.0–50.0)	48.0 (43.0–51.0)	.178
LV end-systolic dimension, mm	28.0 (25.0–31.0)	28.0 (25.0–30.0)	28.0 (25.0–31.0)	27.0 (24.0–31.0)	.841
LV ejection fraction, %	64.1 (57.7–69.5)	64.1 (58.4–69.2)	64.1 (57.7–69.8)	67.0 (56.1–72.8)	.381
Relative wall thickness, mm	0.36 (0.32–0.41)	0.34 (0.31–0.38)	0.38 (0.34–0.42)	0.43 (0.38–0.48)	<.001
LV mass, g	127.7 (103.1–158.8)	112.5 (93.5–137.1)	137.1 (112.1–164.7)	175.6 (136.5–206.1)	<.001
LV mass index, g/m^2^	83.1 (68.9–99.2)	73.3 (62.3–85.6)	89.7 (75.0–103.1)	116.7 (97.0–135.0)	<.001
LA diameter, mm	35.0 (31.0–39.0)	33.0 (30.0–37.0)	36.0 (33.0–40.0)	41.0 (37.0–43.0)	<.001
E wave velocity, m/s	61.0 (50.0–73.0)	68.0 (59.0–78.0)	54.5 (47.0–65.0)	53.0 (43.5–64.5)	<.001
A wave velocity, m/s	60.0 (49.5–75.0)	53.0 (42.0–65.0)	67.0 (55.0–80.0)	71.0 (58.3–84.8)	<.001
Septal e', cm/s	6.9 (5.0–9.1)	9.4 (8.2–11.0)	5.4 (4.5–6.4)	4.4 (3.5–5.4)	<.001
Septal E/e'	8.8 (7.0–11.2)	7.1 (6.0–8.3)	10.4 (8.7–12.2)	12.7 (10.4–14.3)	<.001

Numeric values are expressed as the mean ± standard deviation or median (interquartile range).

ALT, alanine transaminase; AST, aspartate transaminase; GFR, glomerular filtration rate; HDL, high-density lipoprotein; LA, left atrium; LDL, low-density lipoprotein; LV, left ventricle.

**Figure 1:**
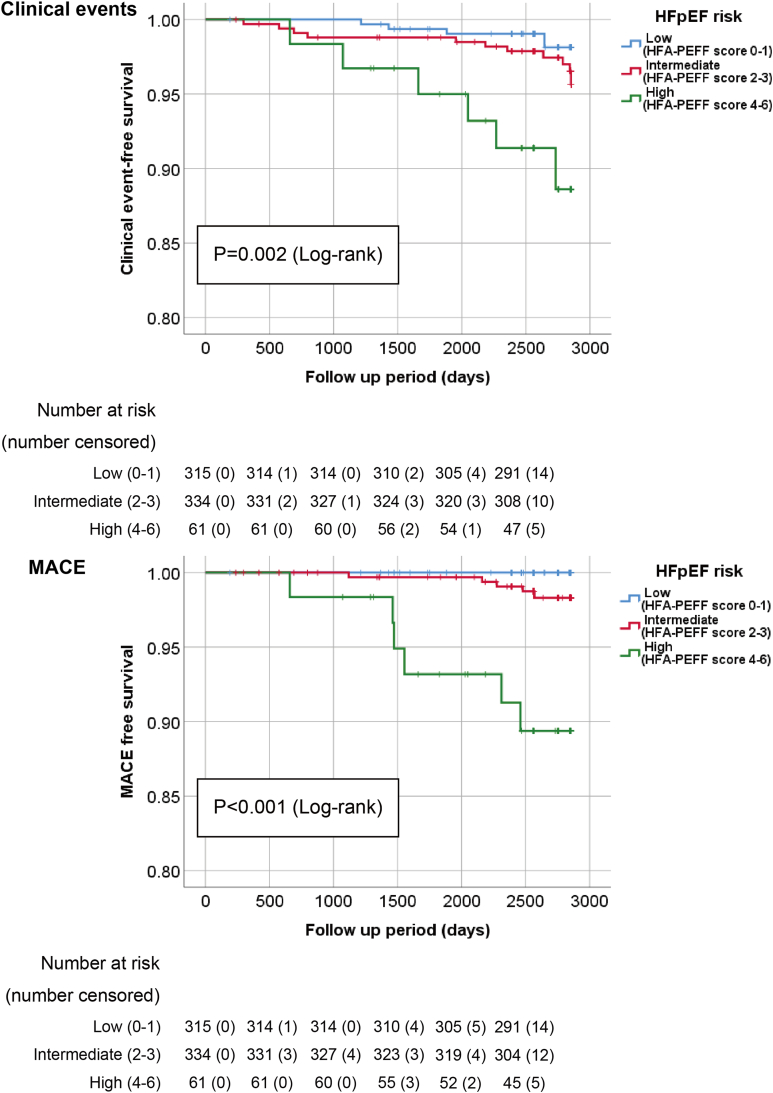
Kaplan–Meier analysis of HFpEF risk for the clinical outcomes. Kaplan–Meier analysis for clinical events (composite endpoint of all-cause mortality and hospitalization due to heart failure) and MACE (composite endpoint of cardiovascular mortality, occurrence of acute myocardial infarction and stroke, hospitalization due to heart failure, and ischemic cardiovascular events) showed that the groups with low and intermediate HFpEF risk had fewer events than the high-risk group (log-rank *P* = .002 and *P* < .001, respectively). MACE, major adverse cardiovascular events; HFpEF, heart failure with preserved ejection fraction.

To assess the clinical significance of HFpEF risk in our subclinical cohort, we examined whether it could be used to stratify clinical prognosis (Figure 1). During the follow-up period of 2753 (2561–2851) days, 21 (3%) participants developed clinical events, including death in 20 patients (95%) and hospitalization for HF in one patient (5%). Furthermore, 11 (2%) patients developed MACE, including acute myocardial infarction in 2 (18%), hospitalization for HF in one (9%), ischemic cardiovascular events in one (18%), and stroke in 7 (64%) participants. The development of liver cancer as a liver-related outcome during this period was observed in one out of 710 subjects (0.1%), who had a high Fib-4 index of 10.14 and a high HFA-PEFF score of 5 at baseline. The Kaplan–Meier life table stratified by HFpEF risk showed that the higher the HFpEF risk group, the higher the rates of clinical events and MACE (log-rank *P* = .002, *P* < .001, respectively). These results suggest the clinical utility of identifying individuals with a high risk of HFpEF in subclinical populations.

### Correlation Between the Fib-4 Index and HFA-PEFF Score

**Figure 2:**
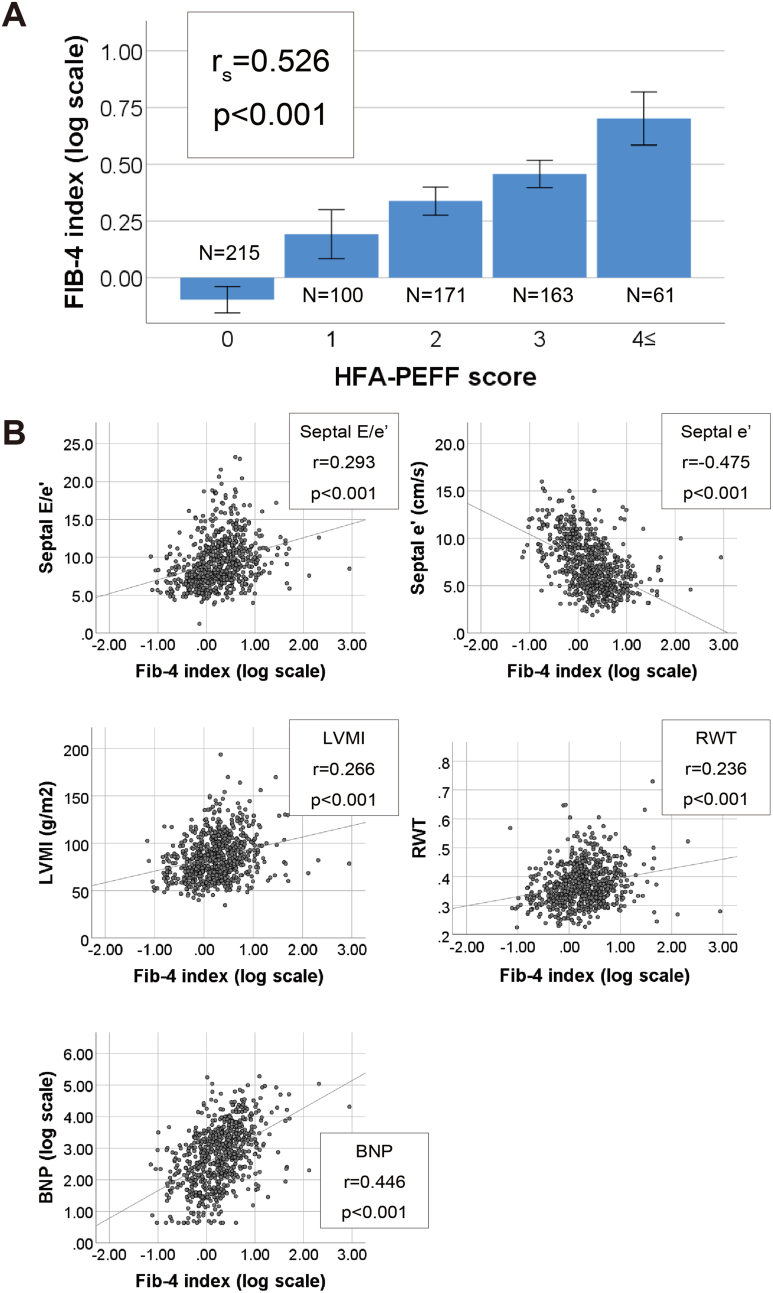
Correlation between the Fib-4 index and HFA-PEFF Score. (A): A significant correlation was found between the Fib-4 index (log scale) and hierarchical modeling of the HFA-PEFF score (r_s_ = 0.526, *P* < .001) using the Spearman’s rank correlation test. Error bars indicate 95% CI. (B): The Fib-4 index (log scale) correlated with parameters for calculating the HFA-PEFF score, including septal E/e’ (r = 0.293, *P* < .001), LVMI (r = 0.266, *P* < .001), RWT (r = 0.236, *P* < .001), and BNP (log scale) (r = 0.446, *P* < .001), and negatively correlated with septal e’ (r = 0.475, *P* < .001) using Pearson's correlation coefficient. BNP, B-type natriuretic peptide; LV, left ventricle; LVMI, LV mass index; RWT, relative wall thickness; CI, confidence interval.

Since the Fib-4 index showed a stepwise variation in HFpEF risk staging (Table 2), we analyzed the association between the Fib-4 index and HFA-PEFF score by correlation analysis using hierarchical modeling of the HFA-PEFF score into 0, 1, 2, 3, and 4–6 points (Figure 2A). Spearman's correlation coefficient revealed a significant correlation between the Fib-4 index (log scale) and hierarchical modeling of the HFA-PEFF score (r_s_ = 0.526, *P* < .001). Moreover, Pearson's correlation coefficient revealed that the Fib-4 index (log scale) correlated with markers associated with other components of the HFA-PEFF diagnostic scoring system (BNP [log scale], r = 0.137, *P* < .001; septal E/e’, r = 0.137, *P* < .001; septal e’, r = 0.137, *P* < .001; LV mass (LVMI), r = 0.097, *P* = .003; RWT, r = 0.152, *P* < .001) (Figure 2B). Furthermore, we examined whether the Fib-4 index predicts high HFpEF risk using multivariate logistic regression analysis, which showed that the Fib-4 index was associated with a high HFpEF risk (N = 61) (Table 3). The results from Model 1 (adjusted for age, sex, and body mass index) showed that an increase in the Fib-4 index was associated with increased odds of high HFpEF risk (aOR, 1.264; 95% CI, 1.059–1.509; *P* = .009), which remained significant in Model 2 (additionally adjusted for regular smoking and alcohol intake, risk factors commonly shared by lifestyle-related diseases; aOR, 1.284; 95% CI, 1.073–1.536; *P* = .006), Model 3 (additionally adjusted for systolic blood pressure, plasma levels of triglycerides, and estimated glomerular filtration rate, which are parameters associated with liver disease, cardiac morphology changes, and plasma BNP levels; aOR, 1.311; 95% CI, 1.078–1.595; *P* = .007), and Model 4 (additionally adjusted for hypertension, dyslipidemia, diabetes mellitus and liver disease, which are major medical histories; aOR, 1.289; 95% CI, 1.061–1.567; *P* = .011). These results suggest that the Fib-4 index is a useful tool for predicting the risk of HFpEF in subclinical individuals.

**Table 3:** Logistic Regression Analysis Examining the Fib-4 Index for Predicting High HFpEF Risk

Fib-4 index	Model 1		Model 2		Model 3		Model 4	
	aOR (95% CI)	*P* Value	aOR (95% CI)	*P* Value	aOR (95% CI)	*P* Value	aOR (95% CI)	*P* Value
Fib-4 index as continuous variables	1.264 (1.059–1.509)	.009	1.284 (1.073–1.536)	.006	1.308 (1.075–1.592)	.007	1.289 (1.061–1.567)	.011
Fib-4 index as categorical variables								
Low (≤ 1.30)	Reference		Reference		Reference		Reference	
Intermediate (1.30–2.67)	1.816 (0.806–4.095)	.150	1.811 (0.802–4.086)	.153	2.489 (1.070–5.793)	.034	2.043 (0.890–4.690)	.092
High (≥ 2.67)	3.054 (1.071–8.709)	.037	3.098 (1.068–8.987)	.037	4.765 (1.560–14.551)	.006	3.203 (1.071–9.581)	.037
P For trend	1.745 (1.038–2.936)	.036	1.759 (1.036–2.989)	.037	2.178 (1.259–3.768)	.005	1.788 (1.045–3.058)	.034

The total number of participants in this study was 710. Model 1 was adjusted for age, sex, and body mass index. Model 2 was adjusted for age, sex, body mass index, regular smoking, and regular alcohol intake. Model 3 was adjusted for age, sex, body mass index, systolic blood pressure, plasma triglyceride levels, and estimated glomerular filtration rate. Model 4 was adjusted for age, sex, body mass index, hypertension, dyslipidemia, diabetes mellitus, and liver disease.

aOR, adjusted odds ratio.

In addition, we examined whether the Fib-4 index could predict high HFpEF risk in a subclinical population using receiver operating characteristic curve analysis. According to the analysis of the area under the curve (AUC), the Fib-4 index was a significant predictor of high HFpEF risk (AUC = 0.782, *P* < .001) (Figure A1). We further verified the cutoff values of the Fib-4 index for the prediction of the HFA-PEFF score by using cutoff values of the Fib-4 index ≤1.30 and ≥2.67 (the negative and positive predictive values of advanced liver fibrosis in NAFLD, respectively).^31^ A Fib-4 index ≤ 1.30 had a sensitivity of 85.2%, while a Fib-4 index ≥ 2.67 had a specificity of 94.8% (Figure A1). These results suggest that the cutoff values of the Fib-4 index for liver fibrosis in NAFLD are also useful for screening for high HFpEF risk. For further confirmation, we examined alternate cutoff values of the Fib-4 index (1.45 low cutoff and 3.25 high cutoff), which are for patients with HIV/hepatitis C virus infection^26^ as reported previously, showing that liver fibrosis can be a risk factor for the development of HFpEF in patients who received clinical care.^32^ A Fib-4 index < 1.45 had a sensitivity of 75.4%, while a Fib-4 index > 3.25 had a specificity of 98.0% (Figure A1).

### Fib-4 Index Allows for Stratification of Prognosis in the Subclinical Population

**Figure 3:**
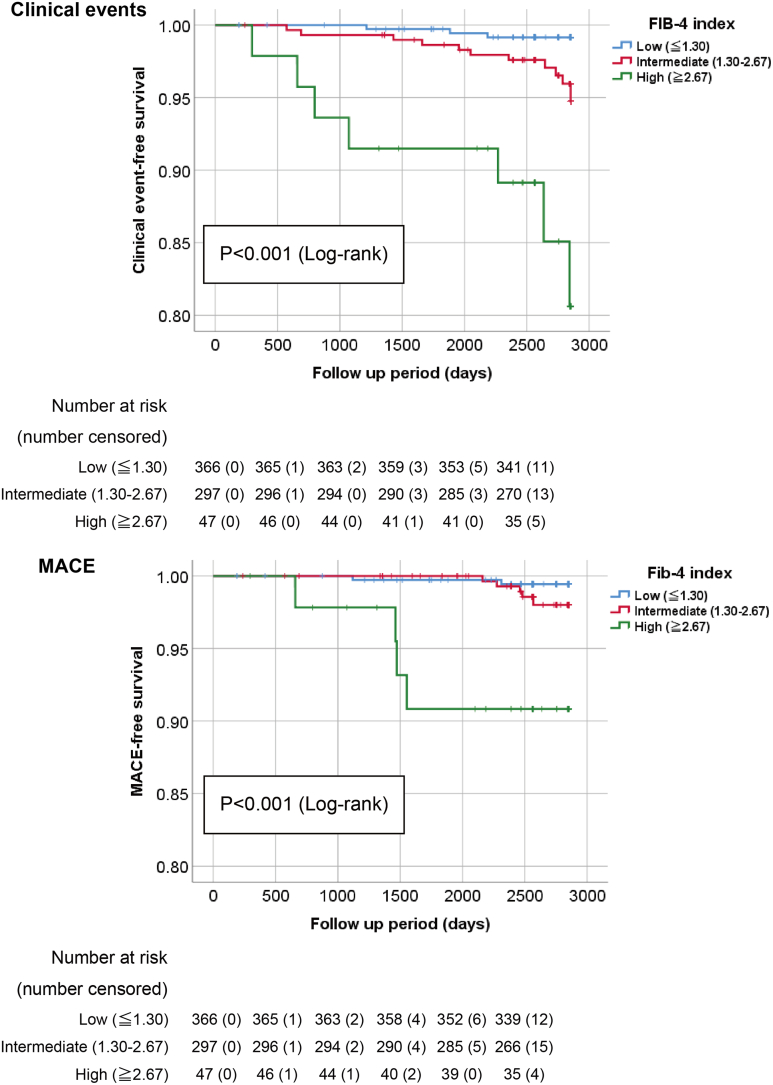
Kaplan–Meier analysis of the Fib-4 Index for the clinical outcomes. Kaplan–Meier analysis for clinical events (composite endpoint of all-cause mortality and hospitalization due to heart failure) and MACE (composite endpoint of cardiovascular mortality, occurrence of acute myocardial infarction and stroke, hospitalization due to heart failure, and ischemic cardiovascular events) showed that groups with low and intermediate Fib-4 index had fewer events than the high score group (both *P* < .001, log-rank). MACE, major adverse cardiovascular events.

Finally, we examined the association between Fib-4 index and clinical prognosis (Figure 3). A total of 366 (52%), 297 (42%), and 47 (7%) participants were classified as having low (≤ 1.30), intermediate (1.30–2.67), and high (≥ 2.67) scores, respectively, using cutoff values of the Fib-4 index of ≤ 1.30 and ≥ 2.67.^31^ The proportion of subjects with a high Fib-4 index in this cohort was similar to that in a previous study (2161 of 29,707 [7%]) based on a large health check-up program.^33^ Using the Kaplan–Meier life table stratified according to these cutoff values, we found that a higher Fib-4 index was associated with higher rates of clinical events and MACE (log-rank *P* < .001) as well as higher HFpEF risk. When we used the alternate cutoff values, 433 (61%), 258 (36%), and 19 (3%) participants were classified as having low (< 1.45), intermediate (1.45–3.25), and high (> 3.25) scores, respectively. Prognostic stratification using the Kaplan–Meier life table of clinical events and MACE was also possible. (log-rank *P* < .001 and *P* = .001, respectively) (Figure A2).

Table 4 provides results of the Cox proportional hazards analyses for predicting clinical events. Univariate analysis revealed that both the Fib-4 index and HFpEF risk were significant predictors of clinical events. Multivariate analysis revealed that they were also significant independent predictors of clinical events using the stepwise method (Model 2): hazard ratio (HR) 1.305 (95% CI, 1.139–1.495; *P* < .001) and HR 1.775 (95% CI, 1.200–2.627; *P* = .004), respectively. When all variables were included in the multivariate model (Model 1), the Fib-4 index remained a significant prognostic predictor. However, the association between the risk of HFpEF and prognosis was weakened, with HR of 1.299 (95% CI, 1.121–1.505; *P* = .001) and 1.415 (95% CI, 0.876–2.286; *P* = .156) (Model 1), respectively, suggesting that the Fib-4 index was strongly associated with both HFpEF risk and prognosis.

**Table 4:** Cox Hazards Regression Analysis for Clinical Events of All-cause Mortality and Hospitalization for Heart Failure

Variables	Univariate		Multivariate Model 1^a^		Multivariate Model 2^b^	
	HR (95% CI)	*P* Value	HR (95% CI)	*P* Value	HR (95% CI)	*P* Value
Age, 1 y	1.084 (1.028–1.144)	.003	1.051 (0.985–1.121)	.136		
Male	2.886 (1.196–6.964)	.018	2.762 (1.127–6.768)	.026	2.659 (1.083–6.531)	.033
Fib-4 index	1.257 (1.128–1.400)	<.001	1.299 (1.121–1.505)	.001	1.305 (1.139–1.495)	<.001
HFpEF risk (HFA-PEFF score)	1.901 (1.289–2.805)	.001	1.415 (0.876–2.286)	.156	1.775 (1.200–2.627)	.004

CI, confidence interval; HR, hazard ratio.

a Model 1: All variables were included in the multivariate model.

b Model 2: Variables were selected using a stepwise method (forward elimination, *P* < .05).

## Discussion

Our findings are as follows. First, the Fib-4 index was significantly associated with HFpEF risk in a subclinical population with undiagnosed HF. Second, both the Fib-4 index and HFpEF risk were independent prognostic factors that allowed prognostic stratification. We emphasize that the present study is the first to demonstrate an association between the Fib-4 index, HFpEF risk (HFA-PEFF score), and prognosis in the subclinical general population.

### Usefulness and Limitation of the HFA-PEFF Scoring System in Health Check-up Programs

The HFA-PEFF score is a diagnostic tool for HFpEF in patients with breathlessness. Its prognostic utility for HF hospitalization or death was demonstrated in a population with unexplained dyspnea and HFpEF in a community-based epidemiologic study.^29^ In this study, we demonstrated that HFpEF risk assessed using the HFA-PEFF score could stratify risk in a subclinical population. Recently, a pilot study reported that HFA-PEFF scores could identify early HFpEF phenogroups.^34^ In this analysis, the HFA-PEFF total score risk categories and biomarkers involved in inflammation and extracellular matrix remodeling were significantly different between the early HFpEF phenogroup and the others.^34^ Thus, the HFA-PEFF score can be a useful tool for stratifying prognosis and identifying early HFpEF in the subclinical population. However, it is difficult to expand the application of the HFA-PEFF score to mass screening, including subjects without breathlessness who attend annual health check-up programs, because echocardiographic examinations require special equipment, such as echocardiographic machines and trained echocardiographers.

### Advantages of the Fib-4 Index in Risk Stratification in Health Check-up Programs

The Fib-4 index is calculated using only 4 parameters that are routinely evaluated in general health checkup programs. This method was originally developed to evaluate liver fibrosis in patients with HIV/hepatitis C coinfection^26^ and then applied to NAFLD.^31^ Although several studies have suggested that NAFLD is associated with HFpEF,^22^^,^^35^ few studies have investigated the usefulness of noninvasive markers of liver fibrosis in the diagnosis of HFpEF in patients with undiagnosed HF. So-Armah et al. reported that advanced liver fibrosis estimated using the Fib-4 index was associated with an increased risk of HFpEF but not HFrEF in patients who received clinical care.^32^ However, this study did not evaluate the relationship between Fib-4 index and cardiac function using echocardiographic measurements. In contrast, our present study had a more comprehensive exploration and demonstrated a clear association between the Fib-4 index, HFA-PEFF score, and prognosis in the subclinical population with undiagnosed HF, and the utility of the Fib-4 index for risk stratification. The cutoff values of the Fib-4 index for both HIV/hepatitis C virus coinfection^26^ and NAFLD^31^ were useful; however, the value for NAFLD may be useful for detecting high-risk for HFpEF, considering the prevalence of these liver diseases in Japan.^30^^,^^36^ Since it can be easily, quickly, and inexpensively measured, routine or repeated measurements of the Fib-4 index could help in selecting preferred candidates for detailed examination of HFpEF risk, which may improve clinical outcomes by diagnosing HFpEF at an early stage.

### Mutual Interaction Between the Liver and Heart

The prevalence of typical NAFLD, as an obesity-related disease, seems to be low in our cohort because they had normal body mass index with few comorbidities, such as diabetes and dyslipidemia. Therefore, a CVP hemodynamic effect, elevated CVP due to passive congestion by HF, should be considered as one of the mechanisms of the association between high Fib-4 index and high risk of HFpEF. Elevated CVP causes hepatocyte atrophy and perisinusoidal edema (ie, liver congestion), which leads to liver stiffness/fibrosis.^17^^,^^18^ Thus, the Fib-4 index in the present study may reflect the liver stiffness/fibrosis associated with potential liver congestion. However, 96% (683 out of 710) of the subjects in our cohort had plasma BNP levels ≤ 80 pg/ml, which can exclude the possibility of elevated CVP due to uncontrolled HF in almost all cases. In addition, stepwise fluctuations in the Fib-4 index were also observed in participants with low HFA-PEFF scores (ie, unlikely to have HFpEF). Therefore, we need to consider the presence of common factors that cause both liver stiffness/fibrosis and cardiac functional/morphological changes. One possibility is undetected hepatic steatosis that can induce low-grade inflammation in both the liver and heart.^37^ A previous cross-sectional study of 8352 subjects who received health check-ups in Japan revealed that the overall prevalence of fatty liver, diagnosed by ultrasonography, was 29.7%.^38^ This prevalence greatly exceeded that of liver disease in our cohort, suggesting the underestimation of fatty liver in our cohort as we did not perform abdominal ultrasonography. Approximately 30% of NAFLD patients in Japan were nonobese, of normal weight, and had significant hepatic fibrosis.^36^ This holds true with regard to HFpEF, as the prevalence of obesity in patients with HFpEF in Japan is lower than that in Western countries,^39^ suggesting a racial difference. Moreover, high salt intake, the leading dietary risk factor for cardiovascular diseases,^40^ is independently associated with an increased risk of fatty liver and advanced liver fibrosis.^41^ The fact that diet-induced hepatic steatosis and inflammation lead to the development of cardiovascular diseases^23^ may underline the importance of evaluating hepatic damage for the prevention of diet-induced cardiovascular diseases. In addition, undiagnosed viral liver disease may also be considered as a factor that affects morphological/functional changes in both the heart and liver, although the prevalence of viral hepatitis in this cohort was comparable to the estimated hepatitis C virus carrier rate in Japan. Although further studies are needed to reveal the interaction between liver and heart function, our results provide valuable insights that are necessary to discover this cardiohepatic interaction to reduce development of HFpEF.

### Study Limitations

This study had several limitations. First, because our study was based on a health check-up program, virological data and examinations to assess the cause of liver disease and fibrosis, detailed echocardiography to assess diastolic function (ie, lateral e', TR velocity, global longitudinal strain , LA volume index) and detailed information on current medications that may affect prognosis and HF symptoms, such as breathlessness, were lacking. Therefore, the prevalence of liver disease may have underestimated potential fatty liver disease. However, the lack of information does not affect our conclusion that high-HFpEF risk candidates who require detailed assessment can be selected from the limited information provided in mass screening. Second, the duration of alcohol consumption, prevalence of autoimmune hepatitis, and development of cirrhosis during the observation period were unknown. Considering that this cohort had a low proportion of heavy alcohol drinkers (2% of the total cohort consumed alcohol > 60 g/d) and that NAFLD is the most prevalent liver disease,^21^^,^^36^ the high Fib-4 index could be reflective of liver fibrosis associated with the progression of undetected hepatic steatosis in many of these participants, rather than alcoholism or other occult liver disease/cirrhotic physiology. Third, this cohort study focused on Japanese rural residents, who may differ in various ways, such as the incidence of hepatic steatosis with nonobese NAFLD, from urban Japanese residents or other races. Finally, there were relatively few clinical events, particularly cardiovascular events. Further studies are required to confirm our findings, particularly regarding HF prognosis.

## Conclusion

The Fib-4 index could be used as a quick, easy, and low-cost screening tool to select candidates who require expert examinations for HFpEF diagnosis from participants in health check-up programs.

## Supplementary Materials

Figures A1 and A2
