# Supplementary material for: Candidate Screening for Heart Failure With Preserved Ejection Fraction Clinic by Fib-4 Index From Subclinical Subjects
Source: Gastro Hep Adv. 2022 Sep 21;2(2):170–81. doi: 10.1016/j.gastha.2022.09.005 (PMC11307393; doi:10.1016/j.gastha.2022.09.005)
Supplement: Figures A1 and A2 [file mmc1.docx]

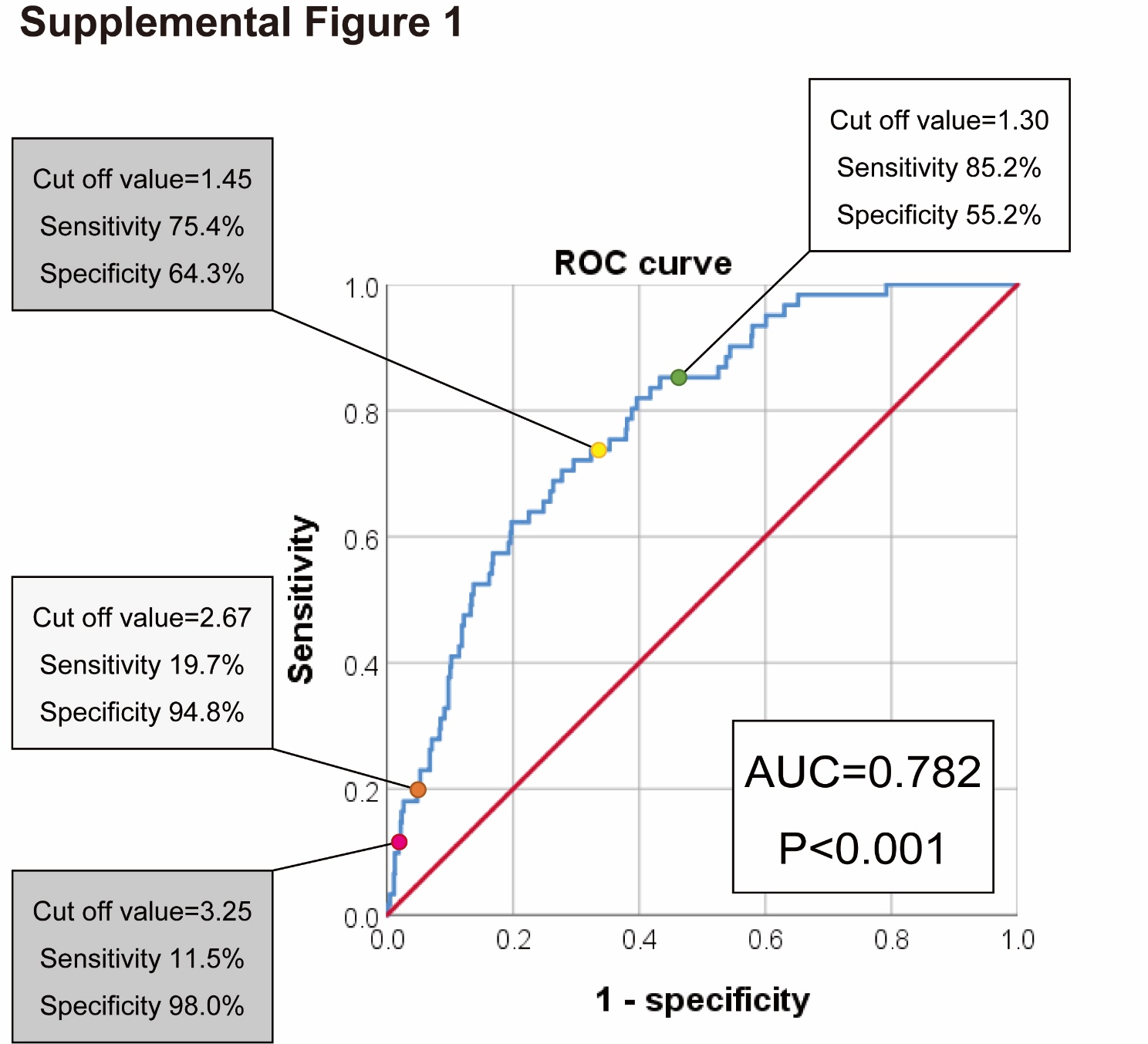


**Supplemental Figure 1. ROC Curve Analysis to Verify the Validity of the Cutoff Value of the Fib-4 Index for Predicting the High Risk of HFpEF (HFA-PEFF Score 4–6 Points).**

On AUC analysis, the Fib-4 index was a significant predictor for predicting the high risk of HFpEF (HFA-PEFF score 4–6 points) (AUC = 0.782, P < 0.001). A Fib-4 index ≤ 1.30, the negative predictive value of liver advanced fibrosis of NAFLD, showed a sensitivity of 85.2%, whereas a Fib-4 index ≥2.67, the positive predictive value of liver advanced fibrosis of NAFLD, showed a specificity of 94.8% (white box). A Fib-4 index <1.45, the negative predictive value of liver advanced fibrosis of viral hepatitis, showed a sensitivity of 75.4%, whereas a Fib-4 index >3.25, the positive predictive value of liver advanced fibrosis of viral hepatitis, showed a specificity of 98.0% (gray box).

AUC, area under the curve; NAFLD, nonalcoholic fatty liver disease; ROC, receiver-operating characteristic.


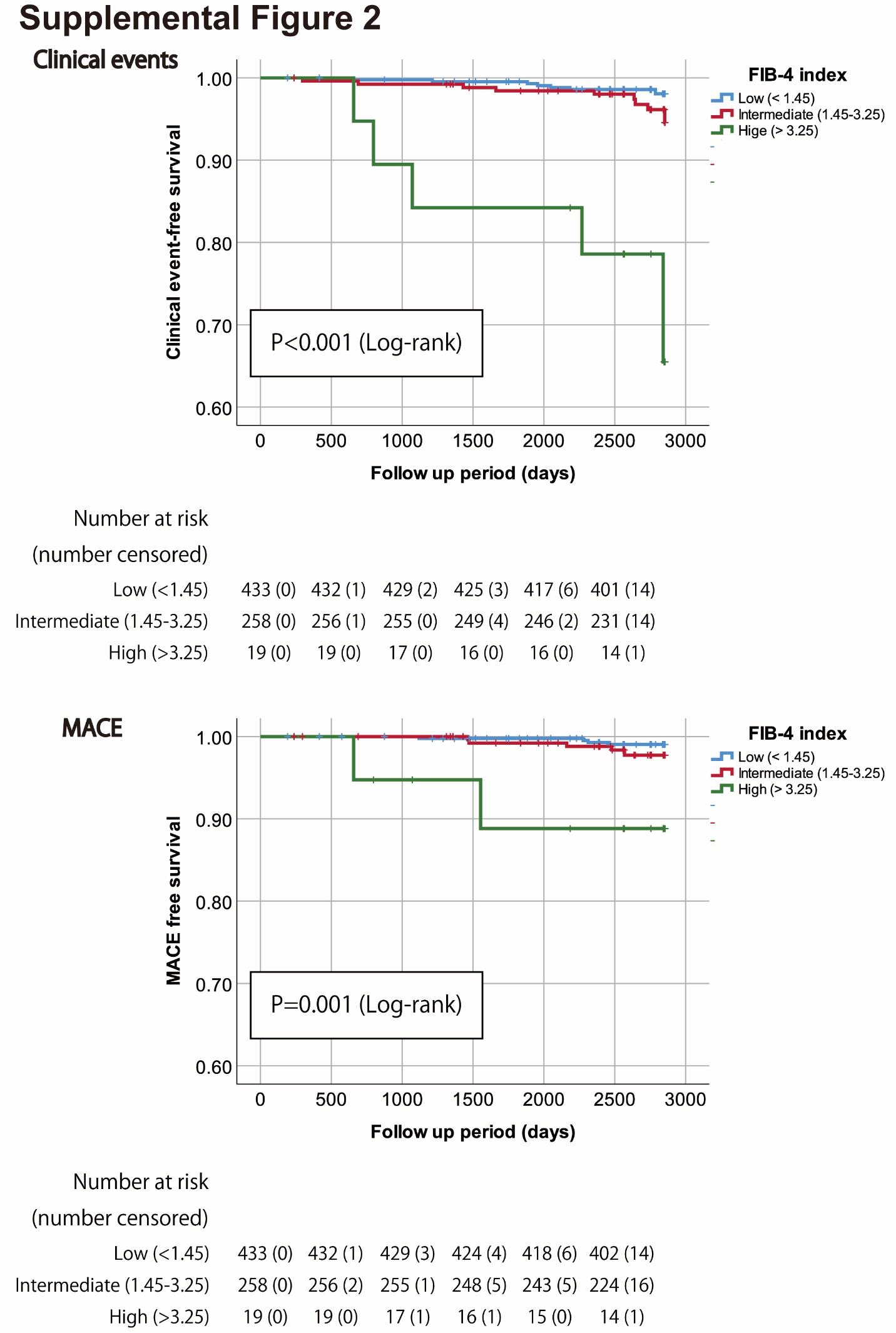


Supplemental Figure 2. Kaplan–Meier Analysis of Another Fib-4 Index Cutoff Value for the Clinical Outcomes.

Kaplan–Meier analysis for the clinical events (composite endpoint of all-cause mortality and hospitalization due to heart failure) and MACE (composite endpoint of cardiovascular mortality, occurrence of acute myocardial infarction and stroke, hospitalization due to heart failure, and ischemic cardiovascular events) showed that the groups with low and intermediate Fib-4 index had fewer events than the high score group using also another cutoff value (1.45 low cut off and 3.25 for the high cutoff) (log-rank P < 0.001, P = 0.001, respectively).

MACE, major adverse cardiovascular events.
